# Can Yellow Stripe Scad Compete with Salmon on Its Role in Platelet Phospholipid Membrane and Its Cardiovascular Benefits?

**DOI:** 10.1155/2019/4929131

**Published:** 2019-07-01

**Authors:** Abdulrahman Yakubu, Azrina Azlan, Su Peng Loh, Sabariah Md Noor

**Affiliations:** ^1^Department of Pathology, Faculty of Medicine and Health Sciences, Universiti Putra Malaysia, 43400 Serdang, Selangor, Malaysia; ^2^Department of Haematology, Faculty of Medical Laboratory Science, Usmanu Danfodiyo University Sokoto, P.M.P 2346, Sokoto, Northern Western, Nigeria; ^3^Department of Nutrition and Dietetics, Faculty of Medicine and Health Sciences, Universiti Putra Malaysia, 43400 Serdang, Selangor, Malaysia

## Abstract

This review article stresses the effective role of dietary fish fillet docosahexaenoic acid (DHA) and eicosapentaenoic acid (EPA) on overweight as a risk factor of cardiovascular disease (CVD) via platelet phospholipid modification. Several reports have demonstrated that saturated fat in overweight evokes systemic inflammation and more importantly predisposes it to cardiovascular disorder. Prospective studies have shown that saturated fat is directly proportional to the level of arachidonic acids (AA), precursor of thromboxane in the platelet phospholipid membrane as omega-6 fatty acid in overweight and obese people. Some literature has demonstrated that omega-3 fatty acid from fish fillet ameliorates inflammation, reduces proinflammatory cytokine, inhibits signaling pathway, and regulates the physical composition of inflammatory leukocytes and free radicals (ROS). Yellow stripe scad (YSS) is a local Malaysian fish that has been shown to contain a comparable level of EPA/DHA content as observed in salmon. This review article will focus on the dietary role of fish fillet that will balance the omega-6 fatty acid/omega-3 fatty acid ratio in platelet phospholipid from YSS to manage and prevent healthy overweight/obesity-related risk factor of CVD and to avoid the risk orthodox drug treatment.

## 1. Introduction

Overweight and obesity are common health issues in many parts of the world. Currently, many countries are experiencing the epidemic burden of overweight and obesity due to change in their lifestyle and low-quality diet. The prevalence is increasing globally according to World Health Organization (WHO) in 2016. According to the National Health and Morbidity Survey (NHMSs) report in 2015, Malaysia experienced a growth in diet-related disorders [1]. Additionally, overweight pandemic in Malaysia was 39% and obesity 13% in adults (aged ≥ 18 years) [2]. Data from NHMSs in 2006, 2011, and 2015 show a systemic increase in overweight and obesity prevalence that cuts across both male and female Malaysian adults aged ≥18 years, 29.1% and 14.5% in 2006 [3] 29.4% and 15.1% in 2011 [4], and 30.0% and 17.7% in 2015, respectively [5]. More recently in 2018, another figure from NHMSs has shown an estimated total percentage of deaths of 74% which comes from noncommunicable diseases (NCDs) largely due to coronary heart disease (CHD) [6]. For type II diabetes, 2.6 million people, that is, 15.2% of Malaysian adults, are diagnosed with diabetes, with 7.2% confirmed and 8.0% not unconfirmed cases. Additionally, 5.8 million Malaysian adults (32.7% of the whole population) are diagnosed with hypertension, with 12.8% confirmed and 19.8% not confirmed cases. Finally, 6.2 million (35.1% of the whole population) Malaysian adults have hypercholesterolemia, with 8.4% confirmed and 26.6% not confirmed cases [5]. The Public Health Institution in 2012 identified overweight and obesity as a risk factor of dyslipidemia with a raised body mass index that predisposes a healthy individual to type-2 diabetes, hypertension, and CVD, which are major life-threatening disorders causing deaths [1, 7]. Prevalence of overweight among adult Malaysians was clearly evidenced between 1996 and 2015. A recent study in Malaysia has shown that the highest overweight level is in Malaysian Indians, followed by Malays, Chinese, and Aborigines [8].

Salmon has been well recognized as the major source of commercial fish oil to treat CVD worldwide, and yet it is often foreign, scarce, expensive, and thus, rarely available. YSS, on the other hand, is a local Malaysian fish that is very abundant, cheap, and available throughout the year in Malaysia; however, the therapeutic effect of YSS on platelet phospholipid is unclear. Thus, this review article seeks to explore the dietary role of YSS as source of EPA/DHA that could harmonize the platelet phospholipid membrane to ameliorate inflammation, oxidative stress, coronary heart disease, and other diet-related disorders in order to avoid the current risk of aspirin and other platelet-related target treatments.

## 2. Platelet Activation, Leptin Resistance, and Inflammation Markers in Overweight- and Obesity-Related CVD

Generally, platelet activation, endothelial dysfunction, and inflammation are factors which contribute to CVD in obese individuals; leptin has also been shown to represent this disorder [9]. Leptin has been implicated in thrombotic occlusion as observed in peripheral arterial disease and stroke [10]. Leptin receptors were identified on the platelet phospholipid membrane [11]. Although its main function is to regulate food, energy, and fat in normal healthy individuals, dysfunctional leptin, in overweight individuals induces leptin resistance to initiate cell signaling that activates the platelet via platelet leptin receptor complex [12]. Activated platelet releases both inflammation and activation markers that could induce the recruitment of monocytes and neutrophils into the endothelial cell matrix [13]. Platelet activation increases the plasma levels of prothrombotic proteins in circulation, which include integrin αIIbβ3, soluble P-selectin, and von Willebrand Factor (vWF) [14]. Integrin *α*IIb*β*3 is the largest cell surface receptor found in platelet ≈80,000 per platelet. High levels of these proteins in obese and overweight individuals are presumed to play a crucial role in facilitating the risk of CVD [15].

## 3. Platelet Activation and Endothelial Cell Interaction in Overweight- and Obesity-Related CVD

The interaction of endothelial cells (ECs) with platelets is central to pathophysiology of leptin resistance observed in overweight/obesity with a low chronic inflammation that facilitates atherothrombosis in EC matrix [16]. This is the initial step of CVD development. The activated platelet first exposes integrin receptor (GPIIb-IIIa), and interaction with expressed VCAM-1 on activated ECs [17] leads to the generation of soluble P-selectin (CD62P) obtained from platelets' *α*-granules that stabilize and maintain the firmed adhesion between the ECs and platelet [18]. Some studies have demonstrated the significant level of sP-selectin in the development of CVD in knockout animals with deficiency of P-selectin that produces a very low atherosclerotic injury in ECs [19]. The expression of disintegrin and metalloproteinase-15 (ADAM-15) by activated ECs facilitates platelet interaction with ECs via platelet integrin receptor (GPIIb-IIIa) [20]. Indeed, in vitro studies have shown that platelet can easily adhere to nonactivated endothelium under shear stress condition [21]. Other studies have emphasized the role of ADAM-15 whose presence on ECs mediates the interaction of platelet GPIIb-IIIa via Arg/Gly/Asp (RGD) sequence to activate the platelets, which in turn release matrix metalloproteinases (MMPs) that further degrade the endothelial grid. These dual actions of ADAM-15 could destabilize and rupture fibronectin layer of atherosclerotic lesion that promotes the generation of platelet thrombi [22]. Thus, activated platelet releases both adhesion and proinflammatory protein from *α*-granules that alter the functional role of ECs; e.g., sCD40L, produced from activated platelet induces the ECs to express the adhesions molecules such as VCAM, E-selectin, and ICAM-1 [23]. sCD40L further facilitates ECs to stimulate the released interleukin-8 (IL-8) and monocyte chemoattractant protein-1 (MCP-1) [23, 24]. The presence of these cytokines in endothelial cell enhances the monocyte recruitments to the site of the endothelial lesion. sCD40L further fosters the expression of MMPs that distort the extracellular molecules. MMPs influence inflammatory reaction at the site of tissue trauma [24]. In addition, activated platelet also liberates IL-1*β*, the main ECs stimulator, to facilitate the expression ICAM-1, including IL-6 and IL-8, in endothelial cell [25]. Platelet factor 4 (PF-4) is also released to aid chemotaxis of monocytes and leukocyte infiltration into the endothelial matrix [26] and further facilitates low-density lipoprotein (LDL) stability by preserving the degradation of LDL receptor in the endothelial matrix [27]. RANTES (regulated activation normal T-cell expressed and secreted) or CCL5 also released from activated platelet is a chemoattractant for monocytes. RANTES and PF-4 are heterophilic in nature; by structural modification of RANTES, RANTES will amplify the effect of monocyte recruitment into the endothelial matrix [26]. The presence of RANTES on the endothelial cell promotes platelet P-selectin [28]. In addition, during platelet activation, platelet-derived growth factor (PDGF) is also released to induce growth of vascular smooth muscles and increases the inner cell mass of endothelial wall. It also assists in the recruitment of monocyte and boosts up the inflammatory response [29]. Thus, all these platelet functions act to produce prostanoid TXA2 from endothelial cell, macrophages, and platelet, which is a potent vasoconstrictor that has been implicated in the pathogenesis of CVD. In endothelial cells, the endothelium-derived prostaglandins such as I2 (PGI2) signal via prostacyclin (IP) receptor, and the interaction between PGI2/IP induces G protein-coupled cAMP and protein kinase A that results in a decreased [Ca^2+^] level, which inhibits platelet activation [30]. However, inhibition of Cox-2 by aspirin via platelet has posed multiple challenges in the treatment of atherosclerosis [31, 32]. Dietary fish fillet with appreciate levels of EPA/DHA intake is known to inhibit platelet activation via harmonization of TXA2 receptor which is the main constituent of the platelet phospholipid membrane and is the first platelet membrane receptor that initiates platelet hyperactive under pathologic event.

## 4. Functional Role of Platelet Phospholipid in Platelet Activation

**Figure 1:**
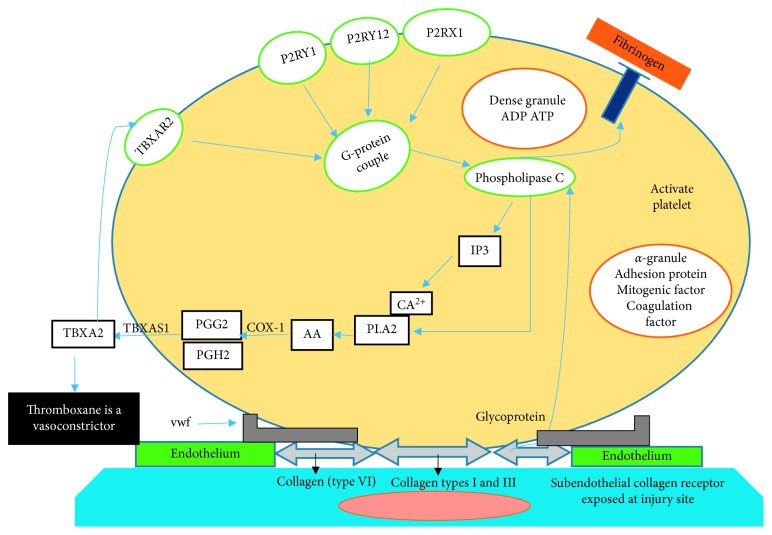
Role of arachidonic acid (AA) released from activated platelet in atherosclerosis.

Platelet phospholipid membrane plays a critical role in several aspects of platelet function. Alterations in phospholipid composition affect platelet activities. Platelet phospholipids release arachidonic acid (AA) via catalysis of phospholipase A2 which is the precursor of platelet prostaglandin synthesis [33]. Under oxidative stress, peripheral arterial blood decreases the speed velocity of platelets by connecting the collagen-bound von Willebrand factor (vWF) and decreases the acceleration by directly interacting with collagen via glycoprotein (GP) receptor complex. The binding of vWF/collagen activates the platelets' collagen receptors which further activate phospholipase C that mediates cascades of reaction pathway (Figure 1) leading to the mobilization of calcium from dense granules [30]. Increased intracellular calcium has been linked with activation of various kinases crucial for structural change, leading to the exposure of the surface of platelet receptors and activating the platelet that mediates the release of granules contents [34]. In fact, the presence of platelet surface procoagulant receptor motivates the activation of coagulation factors that signals a series of zymogen conversions, from prothrombin to thrombin release [35]. Serotonin ADP and ATP are generated from dense platelet granule, and in addition, platelet phospholipids such as phosphatidylcholine and phosphatidylethanolamine are further catalyzed by phospholipase A2 enzymes to release AA [36]. AA activates the release of thromboxane-A2 (TBA2) synthesis, which is the initial stride in platelets' activation. Prostaglandin H2 synthase 1 or cyclooxygenase1 (Cox-1) activates the release of arachidonic acids into cyclic endoperoxides such as PGG2 and PGH2. In platelets, TBXA2 synthase catalyzed PGG2 and PGH2 into TBXA2 [37] (Figure 1).

## 5. Pathophysiology of Platelet Thromboxane's A2 in Overweight/Obesity-Related Coronary Heart Diseases

**Figure 2:**
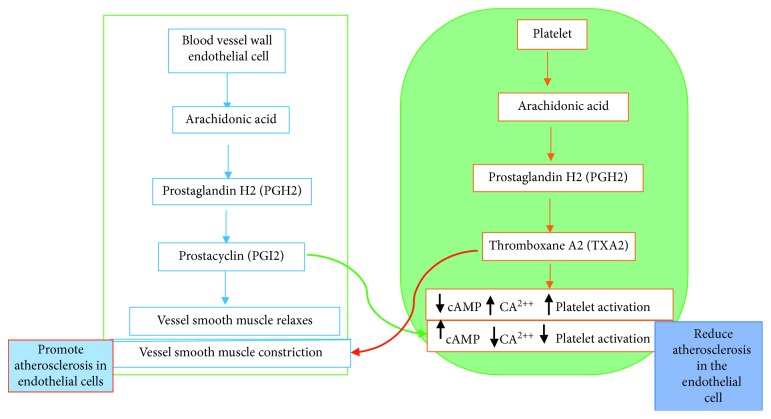
Delicate balance between aggregation/disaggregation and vessel constriction/relaxation.

TXA2 is a good example of platelet agonist. Activated platelet releases significant amounts of TXA2 into the plasma and is thought to be one of the primary causes of thrombotic event in various inflammatory diseases in overweight individuals [16]. However, the presence of endogenous NO and PGI2 serves as potent inhibitors of platelet activity. The impairment in the biogenesis of NO and PGI2 along with excessive availability of TXA2 predisposes overweight and obesity to a thrombotic event. The increased level of TA2 and thromboxane receptor (TP) has been shown to exacerbate hypertension and other overweight/obesity-related risk factors [38]. Therefore, TP receptor inhibitor is a potential factor that helps patients with various inflammatory diseases, induced by overweight and obesity with regard to vascular tone and thrombus formation [39]. Indeed, the current antiplatelet drugs, such as, clopidogrel, aspirin, and ticlopidine, are facing multiple challenges in terms of resistance, bleeding, and gastrointestinal disorders. Hence, there is a need to develop affordable and accessible alternatives to conventional therapies without side effect of aspirin-related drugs in treatment of heart disease. Polyunsaturated fatty acids from fatty fish are metabolized to generate higher levels of TXA3 which is relatively far more less inflammatory than TXA2 and PGI3, thereby shifting the balance toward inhibition of vasoconstriction and platelet aggregation function of TXA2 and PGI3. It is very clear that the shifting will lower the incidence of myocardial infarction (heart attack) and stroke. Perhaps, proinflammatory and vasoconstriction exerted by TXA2 on microvasculature is the probable cause for the genesis of various pathological diseases, such as hepatic inflammation [40], ischemia-reperfusion injury [41], and acute hepatotoxicity [42], among others. The interactions between TXA2 from platelet and prostaglandins such as I2 (PGI2) from ECs (PGI2/TXA2) create a balance to maintain cardiovascular homeostasis, and these have been linked to the benefits of low-dose aspirin, as well as the effect of selective cyclooxygenase-2 (Cox-2) inhibitors on CHD [39] (Figure 2).

## 6. Harmonization of Omega-3 Fatty Acid and Thromboxane (TXA2) Generation in the Absence of Platelet Cox-1 Activities

**Figure 3:**
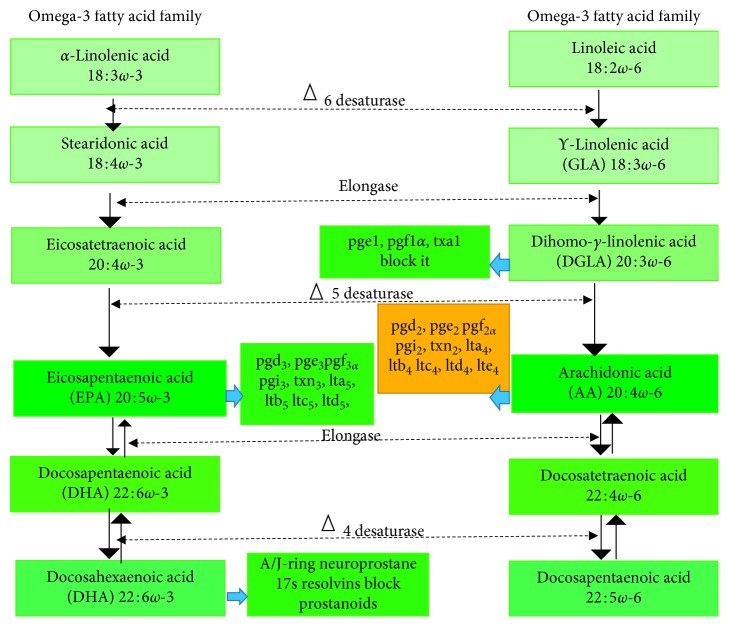
Eicosanoid: prostanglandin = pg, prostacyclin = pgi, thromboxane = txn, leukotrienes = LT, anti-inflammatory = 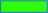, and proinflammation = 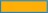.

Several signaling pathways of platelet activation and aggregation have been recognized as major sources of TXA2 and prostaglandin [43]. Omega-6 fatty acid is a platelet phospholipid membrane that generates the release of TXA2 and AA which is the ultimate target of aspirin as Cox-1 [44]. Many studies have shown that platelet has the ability to generate a significant concentration of TXA2 in circulation above the baseline production, making TXA2 an attractive measurement to evaluate platelet activation. Several reports have shown that TXA2 released from activated platelet causes prinzmetal's angina, preeclampsia, and stroke due to its vasoconstriction activities [28]. However, omega-3 fatty acid therapy has been shown to be atheroprotective but not in all studies [45]. Therefore, baseline concentrations of omega-3 fatty acids have an inverse relationship with risk of sudden cardiac death caused by strokes and the mechanism involved in the reduction of TXA2 by omega-3 fatty acid. Three main types of omega-3 fatty acids, as biological active substances, have been recognized, which are *α*-linolenic acid (ALA), eicosapentaenoic acid (EPA), and docosahexaenoic acid (DHA). *α*-Linolenic acid (ALA) can be converted to EPA and DHA enzymatically [46]. Indeed, omega-6 fatty acid is believed to generate multiple copies of TXA2 seen in different thrombotic disorders [33]. However, the harmonization of platelet membrane phospholipids, e.g., omega-6 fatty acid or AA by omega-3 fatty acids, hinders platelet hyperactivity (Figure 3). In addition, the in vitro study of human platelet has demonstrated that omega-3 fatty acids supplementation drastically reduced the TXA2 level and averted the inflammation [47]. This antithrombotic effect of omega-3 fatty acids was confirmed by a study of 62 patients with stable coronary artery disease, and their laboratory evaluation showed omega-3 fatty acid intakes reduce the incidence of coronary artery disease while aspirin resistance was rated 80% in this population. The author further observed a normal stable TXA2 metabolite (urine 11-dehydro thromboxane B2 (UTXB2)) [48]. Thus, putting all these information together, we can suggest that omega-3 fatty acids can reduce TXA2 production via this mechanism of independent pathways of aspirin therapy.

## 7. Dietary Role of Fish Fillet in Overweight- and Obesity-Related Coronary Artery Diseases

The nutritional content of fish depends on its EPA and DHA content, a family of omega-3 fatty acid, that has been shown to play a significant role in blood lipids regulation [49]. A meta-analysis of fish oil intervention and randomized trials showed that omega-3 fatty acid from fatty fish may decrease the mortality rate in patients with CVD [50]. Omega-3 fatty acid may prevent coronary heart disease by reducing serum triacylglycerol (TAG) [51, 52], heart rate [53], serum homocysteine level [54], and blood pressure (BP) [55] and by resolving and regulating multiple inflammatory cells [56] as shown in fish oil supplementation randomized trials. The protective role of omega-3 fatty acid has been shown to be a gradual process, but it protects the individual against mortality and cardiovascular events. Omega-3 fatty acid is also known to protect individuals against some cardiac-arrhythmias at specific doses, to lower hypertension and to alter plasma levels [57]. Fish as a source of high-quality protein is highly nutritious food. It has low saturated fat and is rich in minerals and vitamins. Fish is a very important food that provides 60–70% quality proteins, needed to support cells, tissues, vital organs, and the entire body system in growth, development, body building/repair and to maintain metabolic functions. Multiple studies have highlighted the favorable role of fatty fish as salmon, mackerel herring, and cod as important sources of EPA/DHA. These sources can provide between 800 and 1200 mg of EPA + DHA daily from every 100 g of fish fillet [58]. Currently, EPA and DHA are commercially produced from these sources. The supremacy of salmon as a remarkable source of EPA/DHA lies in the high fat content present in the fish fillet that can provide 1200 mg of EPA/DHA daily [59]. However, salmon is very expensive and is not easily assessable. Recently, three different types of local Malaysian fish have been identified to contain EPA/DHA [60, 61]. These fish are not only cheap but also available throughout the year in Malaysia. According to this data, Yellow stripe scad (selar kuning), Fringescale sardinella (tamban), and Japanese threadfin bream (kerisi) can provide 879, 551, and 436 mg of EPA/DHA per 100 g wet fish fillet, respectively. Of these three species, Yellow stripe scad (YSS) contains the highest EPA/DHA with considerable nutritional value [61].

## 8. Current Available Data on YSS Fish Fillet EPA/DHA

A previous study by Osman et al. shows that out of 100% omega-3 fatty acid content of fish oil extracted from several types of Malaysian fish, only yellow striped scad (YSS) and hardtail pomfret were the two species with the highest percentages of DHA (27.3% and 28.6%, respectively) [62]. Wan Rosli et al. analyzed the fatty acid content of 14 different types of Malaysian fish species, including YSS, and reported that the DHA content across all the 14 different species was significantly higher when compared with the EPA levels [63]. In another study, Hamilton et al. compared the DHA levels between YSS and salmon from previous data of salmon feed (2638 mg/100 g), farmed salmon (1079 mg/100 g), supermarket salmon (969 mg/100 g), and wild salmon (414 mg/100 g) [64]. In these previous data, the DHA value of (629–2633 mg/100 g) from wild salmon and salmon feed, respectively [64], were compared with the current data, and YSS (782.1 mg/100 g wet sample) [61] was found to contain slightly higher DHA content when compared with wild salmon (629 mg/100 g) [64]. This implies that the DHA content of YSS could be comparable to salmon. Recently, Chang et al. confirmed that YSS consumption as fish fillet could increase HDL cholesterol and total cholesterol was significantly higher than baseline in the YSS group in overweight healthy individuals when compared with salmon [65] indicating that YSS could modulate total cholesterol as observed in salmon. This suggests that YSS can be further explored as a potential antithrombotic agent.

## 9. Therapeutic Role of Fish Fillet on Platelet Phospholipid in Overweight/Obesity-Related Cardiovascular Disease

**Figure 4:**
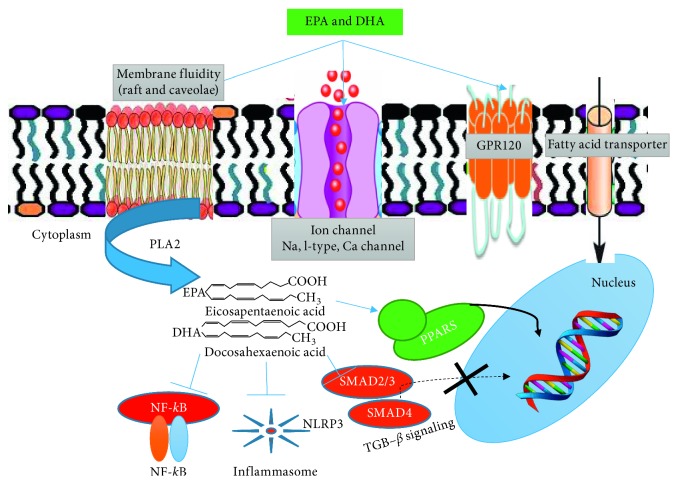
Proposed molecular mechanism of cardioprotection by omega-3 PUFAs. Omega-3 PUFAs modulate platelet cell membrane property when incorporated into the phospholipid bilayer and control membrane ion channels to prevent lethal arrhythmia. Also, omega-3 PUFAs exert anti-inflammatory and antifibrotic effects by modifying NF-*κ*B signaling, the NLRP3 inflammasome, PPAR*α*/*γ*, GPR120, and TGF-*β* signaling (from reference [71]).

Fish fillet contains omega-3 fatty acids that differ from omega-6 fatty acids as arachidonic acids (AA) in the number of double bonds present and the position of the first double bond in relation to the omega-3 fatty acids [66]. For example, docosahexaenoic acid (DHA) has six double bonds and eicosapentaenoic acid (EPA) has five double bonds, both with first double bond located at carbon number three, whereas arachidonic acids or omega-6 fatty has only 4 double bonds at carbon number six [66]. EPA and DHA are 22 carbons long and omega-6 fatty acid or AA is only 20 carbons long. Based on this nomenclature, the formulas AA (20:4n-6), DHA (22:6n-3), and EPA (22:5n-3) are used, respectively. In fact, different enzymes have been identified to recognize each type of fatty acids based on their binding affinities which favor the shift in the balance of the platelet phospholipid membrane toward omega-3 fatty acids to suppress the platelet agonist TXA2 against omega-6 fatty acid, which is a precursor of TXA2 while thromboxane is a potent platelet activator [67, 68]. The saturation of omega-3 fatty acid on the platelet phospholipid membrane is assumed to influence the physical properties of the cell membrane by modifying the microdomain that congregates the lipoprotein in the cell membrane and by functioning as a receptor. Thus, omega-3 fatty acids EPA and DHA are identified as resolvins, protectins, and docosatrienes [69]. The use of fish fillet allows its EPA/DHA content to modulate the platelet phospholipid membrane that regulates the membrane ion channels to prevent lethal arrhythmia [70, 71]. In addition, EPA and DHA are also known to exert anti-inflammatory and antifibrotic effects by altering the NF-*κ*B signaling pathway, PPAR*α*/*γ*, NLRP3 inflammasome, TGF-*β*, and GPR120 signaling [71] (Figure 4).

Indeed, the American Heart Foundation has recommended 250 to 500 mg of EPA/DHA per day for people with no history of coronary artery disease to consume a variety of fatty fish at least twice weekly [72] which can provide the body with the recommended daily need for EPA/DHA [73]. Thus, consumption of certain species of fish frequently will give individuals adequate EPA/DHA necessary to maintain healthy life with no need for additional EPA/DHA from fish oil supplementation (Table 1) [74]. However, consuming fish becomes a problem when its level of mercury contamination is high. Mercury acts as a neurotoxin and can damage the nervous and brain cells. Indeed, despite the multiple health benefits of fatty fish, if the level of mercury contamination is higher, it is advisable to quit such fish consumption on a daily basis (Table 1) [75]. The World Health Organization (WHO) and U.S. Food and Drug Administration (FDA) allow consumption of 0.3 *μ*g of mercury per kg of body weight per day. Also, the Environmental Protection Agency (EPA) in the US has given a lower recommendation of 0.1 *μ*g of mercury per 1 kg of body weight daily [76]. In summary, a 150-pound person should not consume fish fillet with more than 6.8 *μ*g of mercury per day or 48 *μ*g of mercury per week (Table 1).

## 10. Conclusion

Overweight and obesity are preventable inflammatory diseases that can be managed with proper diet and exercise. Well-balanced omega-3 fatty acids, like EPA/DHA, are the most important dietary source from fish fillet that prevents overweight- and obesity-related disorders [50]. Fatty fish has high-quality protein content and low saturated fat and is rich in vitamins and minerals that can contribute 60–70% body proteins and are necessary for growth and development in children, body building, and repair of metabolic functions. A significant source of EPA/DHA is found in fatty fish. Several studies have stressed the influence of 800–1200 mg/day fatty fish such as salmon, herring, cod, and mackerel can provide sufficient EPA and DHA content. Indeed, many commercial EPA/DHA supplements today are produced from these sources. The advantage of salmon as a source of EPA/DHA as compared with other types of fish lies in its high fat and low mercury content [59]. The study by Abd Aziz et al. [61] shows that some local Malaysian fish could provide the daily requirement of these essential minerals and fatty acid contents such as EPA/DHA [60]. The source of these fish is not only cheap but also available throughout the year. These fish are Japanese threadfin bream (kerisi), YSS (selar kuning), and fringescale sardinella (tamban) which can provide 551, 879, and 436 mg of EPA + DHA per 100 g of fish fillet, respectively. Of the three species, YSS contains the highest EPA/DHA with remarkable nutritional content [61]. The biological effects of EPA/DHA are likely due to their bioactive component release into the cellular phospholipid membrane that represses the proinflammatory cytokines and stimulates anti-inflammatory catabolites, such as resolvins and protectins [71]. EPA and DHA have been demonstrated to decrease platelet hypereactivity, via the reorganization of platelet phospholipid membrane, and to reduce the synthesis of thromboxane which is the proaggregatory function platelet, to potentially provide protection against coronary artery disease (CAD) [69]. Indeed, fish fillet from YSS inhibits proinflammatory mediator via the platelet phospholipid membrane and avoids the current side effects of clopidogrel, aspirin, and ticlopidine. This is a remarkable milestone in the history of inflammatory disorders, and fish fillet from YSS could be a potential therapy for pretreatment of various inflammatory diseases such as hypertension, type-2 diabetes, cancer, and cardiovascular-related diseases.

## Figures and Tables

**Table 1:** EPA/DHA levels in different species of fish and their mercury contamination (adopted from Omega3HealthElite Premium Omega-3 Fish Oil Supplement-High).

Fish species and description	DHA + EPA mg per 100 g	Mercury level in *μ*g	Contamination rating	Fish categories
Salmon, Atlantic, cooked	1840	4	Low	Best choice
Salmon coho, farmed, cooked	1279	4	Low	Best choice
Anchovy, European, canned in oil	2055	3.1	Low	Best choice
Oyster, Pacific, cooked	1376	2.2	Low	Best choice
Sardine, Atlantic, canned in oil	982	2.2	Low	Best choice
Pollock, Atlantic, cooked	542	5.64	Low	Best choice
Flatfish (flounder and sole species), cooked	501	2.18	Low	Best choice
Salmon, coho, wild, cooked	1059	4	Low	Best choice
Catfish, channel, wild, cooked	237	4.55	Low	Best choice
Haddock, cooked	238	10	Medium	Best choice
Trout, cooked	1154	12.9	Medium	Best choice
Smelt, rainbow, cooked	889	14.7	Medium	Best choice
Herring, Pacific, cooked	2125	15.3	Medium	Best choice
Mackerel, Pacific (chub), cooked	1848	16	Medium	Best choice
Spiny lobster, cooked	480	16.9	Medium	Best choice
Sheepshead, cooked	190	16.9	Medium	Best choice
Carp, cooked	451	20	Medium	Best choice
Cod, Pacific, cooked	276	20.2	Medium	Best choice
Lingcod, cooked	263	20.2	Medium	Best choice
Ocean perch, Atlantic, cooked	374	22	Medium	Best choice
Tuna, white, canned	862	23.3	Medium	Good choice
Tilefish, cooked, Atlantic	905	26.2	Medium	Good choice
Bass, striped, cooked	967	27.7	Medium	Good choice
Mackerel, Atlantic, cooked	1203	33.1	High	Best choice
Halibut, Greenland, cooked	1178	43.9	High	Good choice
Halibut, Atlantic and Pacific, cooked	465	43.9	High	Good choice
Tuna, yellowfin, fresh, cooked	279	64.4	High	Good choice
Sablefish, cooked	1787	65.7	High	Good choice
Bluefish, cooked	988	67	High	Good choice
Tuna, fresh, bluefin and bigeye, cooked	1504	125.4	Very high	Avoid
Mackerel, king, cooked	401	132.9	Very high	Avoid
Swordfish, cooked	819	181.1	Very high	Avoid
Tilefish, Gulf of Mexico, cooked	905	263.9	Very high	Avoid
